# TRIM5α Restriction of HIV-1-N74D Viruses in Lymphocytes Is Caused by a Loss of Cyclophilin A Protection

**DOI:** 10.3390/v14020363

**Published:** 2022-02-10

**Authors:** Anastasia Selyutina, Lacy M. Simons, Karen A. Kirby, Angel Bulnes-Ramos, Pan Hu, Stefan G. Sarafianos, Judd F. Hultquist, Felipe Diaz-Griffero

**Affiliations:** 1 Department of Microbiology and Immunology, Albert Einstein College of Medicine, Bronx, NY 10461, USA; anastasia.selyutina@einsteinmed.edu (A.S.); angelbulnesramos@gmail.com (A.B.-R.); pan.hu@einsteinmed.edu (P.H.); 2 Division of Infectious Diseases, Northwestern University Feinberg School of Medicine, Chicago, IL 60611, USA; lacy.simons@northwestern.edu (L.M.S.); judd.hultquist@northwestern.edu (J.F.H.); 3 Laboratory of Biochemical Pharmacology, Department of Pediatrics, Emory University School of Medicine, Atlanta, GA 30322, USA; karen.kirby@emory.edu (K.A.K.); stefanos.sarafianos@emory.edu (S.G.S.); 4 Children’s Healthcare of Atlanta, Atlanta, GA 30322, USA

**Keywords:** HIV-1, N74D, CPSF6, TRIM5α_hu_, capsid, core, restriction

## Abstract

The core of HIV-1 viruses bearing the capsid change N74D (HIV-1-N74D) do not bind the human protein CPSF6. In primary human CD4^+^ T cells, HIV-1-N74D viruses exhibit an infectivity defect when compared to wild-type. We first investigated whether loss of CPSF6 binding accounts for the loss of infectivity. Depletion of CPSF6 in human CD4^+^ T cells did not affect the early stages of wild-type HIV-1 replication, suggesting that defective infectivity in the case of HIV-1-N74D viruses is not due to the loss of CPSF6 binding. Based on our previous result that cyclophilin A (Cyp A) protected HIV-1 from human tripartite motif-containing protein 5α (TRIM5α_hu_) restriction in CD4^+^ T cells, we found that depletion of TRIM5α_hu_ in CD4^+^ T cells rescued the infectivity of HIV-1-N74D, suggesting that HIV-1-N74D cores interacted with TRIM5α_hu_. Accordingly, TRIM5α_hu_ binding to HIV-1-N74D cores was increased compared with that of wild-type cores, and consistently, HIV-1-N74D cores lost their ability to bind Cyp A. In agreement with the notion that N74D capsids are defective in their ability to bind Cyp A, we found that HIV-1-N74D viruses were 20-fold less sensitive to TRIMCyp restriction when compared to wild-type viruses in OMK cells. Structural analysis revealed that N74D hexameric capsid protein in complex with PF74 is different from wild-type hexameric capsid protein in complex with PF74, which explains the defect of N74D capsids to interact with Cyp A. In conclusion, we showed that the decreased infectivity of HIV-1-N74D in CD4^+^ T cells is due to a loss of Cyp A protection from TRIM5α_hu_ restriction activity.

## 1. Introduction

Human cleavage and polyadenylation specificity factor subunit 6 (CPSF6) is a nuclear protein that belongs to the serine/arginine-rich protein family. Expression of a cytosolic fragment of CPSF6 [CPSF6(1–358)] was found to potently block human immunodeficiency virus-1 (HIV-1) infection before the formation of 2-long terminal repeat circles [1], and this inhibition of HIV-1 infection correlated with the ability of CPSF6(1–358) to bind to the capsid and prevent uncoating [2,3,4]. The serial passaging of HIV-1 in human cells overexpressing CPSF6(1–358) resulted in the generation of escape-mutant viruses bearing the N74D capsid change (HIV-1-N74D) [1], and binding studies of HIV-1 capsids with N74D mutations to CPSF6(1–358) demonstrated a lack of binding as the mechanism for escape [1,5]. HIV-1-N74D viruses have decreased infectivity in Jurkat T cells when compared to wild-type, and recent studies showed that TRIM34 is important for this phenotype [6]. Although the overexpression of full-length CPSF6 remained nuclear and did not block HIV-1 infection, these experiments functionally linked CPSF6 to the HIV-1 capsid. Knockdown or knockout of human CPSF6 expression in different human cell lines did change HIV-1 integration site selection [5,7,8,9,10]. Several reports have also suggested that full-length CPSF6 may facilitate the entry of the virus core into the nucleus [10,11,12]. The lack of a correlation between the loss of CPSF6 binding to HIV-1 and decreased infectivity in human cell lines indicates that cell-type specific differences in the pathways surrounding early replication contribute to these discrepancies, suggesting the need for additional work using human primary cell models [13,14,15,16].

This work used human primary cells to examine the role of CPSF6 in HIV-1 replication. Our experiments demonstrated that the HIV-1 capsid mutations N74D or A77V affected the capsid’s ability to interact with CPSF6, as displayed by infectivity phenotypes in human primary peripheral blood mononuclear cells (PBMCs) and CD4^+^ T cells. When compared with the wild-type virus, HIV-1-N74D showed decreased infectivity, but HIV-1-A77V infectivity was less affected. These different mutant virus infectivity phenotypes suggested that the reduced primary cell infectivity observed in the case of HIV-1-N74D was not likely to be due to a CPSF6-binding defect. To test this hypothesis directly, we challenged CPSF6-depleted human primary CD4^+^ T cells with wild-type HIV-1-N74D and HIV-1-A77V viruses. Remarkably, depletion of CPSF6 did not affect HIV-1 infection, suggesting that the loss of capsid-CPSF6 interactions did not account for the observed decrease in infectivity by the mutants. One possibility is that a different protein may be responsible for reduced HIV-1-N74D infectivity. Recently, we and others have demonstrated that cyclophilin A (Cyp A) protects the HIV-1 core from restriction by the human tripartite motif-containing protein 5α TRIM5α_hu_ in primary CD4^+^ T cells [17,18] To test whether TRIM5α_hu_ is involved in decreased HIV-1-N74D infectivity, we challenged TRIM5α_hu_-depleted human primary CD4^+^ T cells with HIV-1-N74D. Interestingly, we observed that TRIM5α_hu_ depletion rescued HIV-1-N74D infectivity, suggesting that TRIM5α_hu_ is responsible for the restriction observed in human primary T cells. In light of the role of Cyp A to protect the core from restriction by TRIM5α_hu_, we also tested the ability of the N74D mutation-containing capsids to bind to Cyp A. We found that these capsids lost their ability to interact with Cyp A, which may explain the reason that HIV-1-N74D is restricted by TRIM5α_hu_. Overall, our results show that the HIV-1-N74D mutant virus is restricted by TRIM5α_hu_ due to an inability to bind Cyp A.

## 2. Materials and Methods

### 2.1. Cell Lines and Drugs

Human A549 and 293T cells, owl monkey kidney OMK cells and canine Cf2Th cells obtained from the American Type Culture Collection (ATCC) were grown at 37 °C in Dulbecco’s Modified Eagle Medium (DMEM) supplemented with 10% FBS and 1% penicillin-streptomycin. Human Jurkat cells obtained from the American Type Culture Collection (ATCC) were grown at 37 °C in Roswell Park Memorial Institute Medium (RPMI) supplemented with 10% FBS and 1% penicillin-streptomycin. PF74 and cyclosporine A were obtained from Sigma Aldrich (SML0835). They were dissolved in dimethyl sulfoxide (DMSO) to create a stock of 10 mM. 

### 2.2. Creation of Stable Cell Lines Expressing Different Proteins

Retroviral vector encoding an artificial trimeric-Cyp A construct (GCN4-TriL2-Cyp) tagged with an influenza hemagglutinin (HA) at the C terminus was created using the pLPCX vector [19]. Recombinant viruses were produced in 293T cells by co-transfecting pLPCX plasmids with pVPack-GP and pVPack-VSV-G packaging plasmids (Stratagene). The pVPack-VSV-G plasmid encodes the vesicular stomatitis virus (VSV) G envelope glycoprotein, which allows efficient entry into a wide range of vertebrate cells. Canine Cf2Th cells were transduced and selected in the appropriate concentration of puromycin (1–5 μg/mL).

### 2.3. Fate of the Capsid Assay

Fate of the capsid assay was performed as described [20]. Human A549 cells were infected with wild-type HIV-1-GFP or mutant HIV-1-N74D-GFP, normalized to p24, with 10 µM PF74, 10 µM Cyp A, or DMSO solvent. After incubation at 37 °C for 16 h, cells were detached with 7 mg/mL Pronase for 5 min on ice and washed three times with ice-cold PBS. Cell pellets were resuspended in hypotonic buffer (10 mM Tris-HCl, pH 8.0, 10 mM KCl, 1 mM EDTA) and incubated for 20 min on ice. Cells were lysed in a 7.0 mL Dounce homogenizer with pestle B. The cellular debris and the nuclear fraction were removed by centrifugation for 7 min at 3000 rpm. The supernatant fraction was layered onto a 50% sucrose (weight:volume) cushion in 1x PBS and centrifuged at 125,000× *g* for 2 h at 4 °C in a Beckman SW41 rotor. Input, soluble, and pellet fractions were analyzed by Western blotting using anti-p24 antibody. 

### 2.4. Infection Using HIV-1-GFP Reporter Viruses 

Recombinant HIV-1 strains (e.g., HIV-1-N74D and HIV-1-A77V) expressing GFP, and pseudo-typed with VSV-G, were prepared as previously described [21]. All viruses were titered according to p24 levels and infectivity. Viral challenges were performed in 24-well plates by infecting 50,000 cells (PBMCs or CD4^+^ T cells) per well. Infectivity was determined by measuring the percentage of GFP-positive cells using flow cytometry (BD FACSCelesta, San Jose, CA, USA).

### 2.5. Capsid Expression and Purification 

The pET-11a vector was used to express HIV-1 capsid proteins containing the A14C and E45C mutations. Point mutations N74D and A77V were introduced using the QuikChange II site-directed mutagenesis kit (Stratagene) according to the manufacturer’s instructions. All proteins were expressed in *Escherichia coli* one-shot BL21star (DE3) cells (Invitrogen, Carlsbad, CA, USA), as previously described [22]. Briefly, cells were inoculated in Luria-Bertani medium and cultured at 30 °C until mid-log phase (Absorbance at 600 nm, 0.6–0.8). Protein expression was induced with 1 mM isopropyl-β-d-thiogalactopyranoside overnight at 18 °C. Cells were harvested by centrifugation at 5000*× g* for 10 min at 4 °C, and pellets were stored at −80 °C until purification. Purification of capsids was carried out as follows. Pellets from two-liter cultures were lysed by sonication (Qsonica microtip: 4420; A = 45; 2 min; 2 sec on; 2 sec off for 12 cycles), in 40 mL of lysis buffer (50 mM Tris pH = 8, 50 mM NaCl, 100 mM β-mercaptoethanol, and Complete ethylenediaminetetraacetic acid (EDTA)-free protease inhibitor tablets). Cell debris was removed by centrifugation at 40,000*× g* for 20 min at 4 °C. Proteins from the supernatant were precipitated by incubation with one-third the volume of saturated ammonium sulfate containing 100 mM β-mercaptoethanol for 20 min at 4 °C, and centrifugation at 8000*× g* for 20 min at 4 °C. Precipitated proteins were resuspended in 30 mL of buffer A (25 mM 2-(N-morpholino) ethanesulfonic acid (MES), pH 6.5, and 100 mM β-mercaptoethanol) and sonicated 2–3 times (Qsonica microtip: 4420; A = 45; 2 min; 1 sec on; 2 sec off). The protein sample was dialyzed three times in buffer A (2 h, overnight, and 2 h), sonicated, diluted in 500 mL of buffer A, and then separated sequentially on a 5-mL HiTrap Q HP column followed by a 5-mL HiTrap SP FF column (GE Healthcare), which were both pre-equilibrated with buffer A. Capsid proteins were eluted from the HiTrap SP FF column using a linear gradient of concentrations ranging from 0–2 M NaCl. The eluted fractions that had the highest protein levels were selected based on absorbance at 280 nm. Pooled fractions were dialyzed three times (2 h, overnight, and 2 h) in storage buffer (25 mM MES, 2 M NaCl, 20 mM β-mercaptoethanol). Samples were concentrated to 20 mg/mL using Centriprep Centrifugal Filter Units and stored at –80 °C.

### 2.6. Assembly of Stabilized HIV-1 Capsid Tubes 

One milliliter of monomeric capsid (5 mg/mL) was dialyzed in SnakeSkin dialysis tubing (10K MWCO, Thermo Scientific, Waltham, MA, USA) using a buffer that was high in salt and contained a reducing agent (Buffer 1: 50 mM Tris, pH 8, 1 M NaCl, 100 mM β-mercaptoethanol) at 4 °C for 8 h. The protein was then dialyzed using the same buffer without the reducing agent β-mercaptoethanol (Buffer 2: 50 mM Tris, pH 8, 1 M NaCl) at 4 °C for 8 h. The absence of β-mercaptoethanol in the second dialysis allowed the formation of disulfide bonds between Cysteine 14 of one monomer and the Cysteine 45 of a second monomer in the hexamer. Finally, the protein was dialyzed using Buffer 3 (20 mM Tris, pH 8, 40 mM NaCl) at 4 °C for 8 h. Assembled complexes were kept at 4 °C for up to 1 month.

### 2.7. Capsid Binding Assay Protocol

Human HEK293T cells were transfected for 24 h with a plasmid expressing the protein of interest (TRIM5α_hu_). The culture media was completely removed and cells were scraped off the plate and lysed in 300 μL of capsid binding buffer (CBB: 10 mM Tris, pH 8, 1.5 mM MgCl_2_, 10 mM KCl). Cells were rotated for 15 min at 4 °C and then centrifuged to remove cellular debris (21,000*× g*, 15 min, 4 °C). Cell lysates were incubated with stabilized HIV-1 capsid tubes for 1 h at 25 °C. The stabilized HIV-1 capsid tubes were then centrifuged at 21,000*× g* for 2 min. Pellets were washed 2–3 times by resuspension and centrifugation in CBB or phosphate-buffered saline (PBS). Pellets were resuspended in 1× Laemmli buffer and analyzed by western blotting using an anti-p24 antibody and other appropriate antibodies.

### 2.8. Preparation of PBMCs and CD4^+^ T Cells

PBMCs from healthy-donor whole blood were isolated by density gradient centrifugation using Ficoll-Paque Plus (GE Health Care, Chicago, IL, USA). Whole blood (40 mL) was centrifuged at 300*× g* for 10 min, and the plasma layer was removed and replaced with Hank’s Balanced Salt solution (HBSS; Sigma Aldrich, St. Louis, MO, USA). The blood sample was then diluted 1:2 with HBSS, and 20 mL of the diluted sample was layered on top of 20 mL Ficoll-Paque Plus and centrifuged at 300*× g* for 30 min. The resulting buffy coat layer was collected, washed twice with HBSS, and resuspended in Roswell Park Memorial Institute (RPMI) medium containing 10% (*vol*/*vol*) fetal bovine serum (FBS) and 1% (*vol*/*vol*) penicillin-streptomycin, and activated with IL-2 (100 U/mL) (Human IL-2; Cell Signaling Technology, #8907SF) and phytohemagglutinin (1 µg/mL) for 3 days. CD4^+^ T cells were obtained via negative selection from PBMCs using a human CD4^+^ T-cell isolation kit (MACS Miltenyi Biotec, #130-096-533, Bergisch Gladbach, Germany). PBMCs (1 × 10^7^ total cells) were resuspended in 40 µL of CD4^+^ T-cell isolation buffer (PBS, pH 7.2, 0.5% bovine serum albumin (BSA), and 2 mM EDTA). CD4^+^ T-cell biotin-antibody cocktail (10 µL) was then added to the PBMCs and incubated at 4 °C for 5 min. CD4^+^ T-cell isolation buffer (30 µL) and CD4^+^ T-cell microbead cocktail (20 µL) were then added and further incubated for 10 min at 4 °C. Depending on the number of PBMCs isolated, either an LS or MS column attached to a Magnetic Activated Cell Sorting Separator was prewashed using 3 mL or 6 mL of ice-cold CD4^+^ T cell isolation buffer, respectively. The PBMC suspension was added to the column and the flow-through was collected in a 15 mL tube. The LS or MS column was then washed (3 mL or 6 mL, respectively, with ice-cold CD4^+^ T-cell isolation buffer), and the flow-through was collected. The newly isolated CD4^+^ T cells were then centrifuged at 800*× g* for 5 min and resuspended in RPMI medium supplemented with IL-2 (100 U/mL).

### 2.9. CRISPR-Cas9 Knockouts in Primary CD4^+^ T Cells

Detailed protocols for the production of CRISPR-Cas9 ribonucleoprotein complexes (crRNPs) and primary CD4^+^ T-cell editing have been previously published [23,24]. Briefly, lyophilized CRISPR RNA (crRNA) and trans-activating CRISPR RNA (tracrRNA; Dharmacon, Lafayette, CO, USA) were each resuspended at a concentration of 160 µM in 10 mM Tris-HCl (pH 7.4), and 150 mM KCl. Five microliters of 160 µM crRNA were then mixed with 5 µL of 160 µM tracrRNA and incubated for 30 min at 37 °C. The gRNA:tracrRNA complexes were then mixed gently with 10 µL of 40 µM purified Cas9-NLS protein (UC-Berkeley Macrolab) to form crRNPs. Complexes were aliquoted and frozen in 0.2-mL PCR tubes (USA Scientific, Ocala, FL, USA) at −80 °C until further use. crRNA guide sequences used in this study were a combination of sequences derived from the Dharmacon predesigned Edit-R library for gene knockouts, and custom-ordered sequences as indicated (Table 1).

PBMCs were isolated by density gradient centrifugation using Ficoll-Paque Plus (GE Health Care, #17-1440-02). PBMCs were washed thrice with 1× PBS to remove platelets and resuspended at a final concentration of 5 × 10^8^ cells/mL in 1× PBS, 0.5% BSA, and 2 mM EDTA. Bulk CD4^+^ T cells were subsequently isolated from PBMCs by magnetic negative selection using an EasySep Human CD4^+^ T Cell Isolation Kit (STEMCELL, per manufacturer’s instructions). Isolated CD4^+^ T cells were suspended in complete RPMI medium, consisting of RPMI-1640 (Sigma Aldrich) supplemented with 5 mM 4-(2-hydroxyethyl)-1-piperazineethanesulfonic acid (Corning, Corning, NY, USA), 50 μg/mL penicillin/streptomycin (Corning), 5 mM sodium pyruvate (Corning), and 10% FBS (Gibco). Media was supplemented with 20 IU/mL IL-2 (Miltenyi) immediately before use. For activation, bulk CD4^+^ T cells were immediately plated on anti-CD3-coated plates (coated for 12 h at 4 °C with 20 µg/mL anti-CD3 antibody (UCHT1, Tonbo Biosciences)) in the presence of 5 µg/mL soluble anti-CD28 antibody (CD28.2, Tonbo Biosciences). Cells were stimulated for 72 h at 37 °C in a 5%-CO_2_ atmosphere prior to electroporation. After stimulation, cell purity and activation were verified by CD4/CD25 immunostaining and flow cytometry as previously described [23].

After three days of stimulation, cells were resuspended and counted. Each electroporation reaction consisted of between 5 × 10^5^ and 1 × 10^6^ T cells, 3.5 µL RNPs, and 20 µL of electroporation buffer. The crRNPs were thawed to room temperature. Immediately prior to electroporation, cells were centrifuged at 400*× g* for 5 min, the supernatant was removed by aspiration, and the pellet was resuspended in 20 µL of room temperature P3 electroporation buffer (Lonza, Basel, Switzerland) per reaction. Cell suspensions (20 µL) were then gently mixed with each RNP and aliquoted into a 96-well electroporation cuvette for nucleofection with the 4D 96-well shuttle unit (Lonza) using pulse code EH-115. Immediately after electroporation, 100 µL of prewarmed media without IL-2 was added to each well and cells were allowed to rest for 30 min in a cell-culture incubator at 37 °C. Cells were subsequently moved to 96-well flat-bottomed culture plates prefilled with 100 µL warm complete media with IL-2 at 40 U/mL (for a final concentration of 20 U/mL) and anti-CD3/anti-CD2/anti-CD28 beads (T cell Activation and Stimulation Kit, Miltenyi) at a 1:1 bead:cell ratio. Cells were cultured at 37 °C in a 5%-CO_2_ atmosphere, dark, humidified cell-culture incubator for four days to allow for gene knockout and protein clearance, with additional media added on day 2. To check knockout efficiency, 50 µL of mixed culture was transferred to a centrifuge tube. Cells were pelleted, the supernatant removed, and the pellets were resuspended in 100 µL 2.5× Laemmli Sample Buffer. Protein lysates were heated to 98 °C for 20 min before storage at –20 °C until assessment by western blotting.

### 2.10. Crystallization, Data Collection, and Structure Determination

Full-length HIV-1 CA containing an N74D mutation in a pET11a vector was expressed and purified as previously described [25]. Hexagonal N74D CA apo crystals grew in conditions as previously described for WT CA [26]. N74D CA apo crystals were cryoprotected briefly in a solution containing 20% glycerol before flash freezing in liquid nitrogen. Some N74D CA apo crystals were soaked in a solution containing PF74 for ~4 h, then transferred briefly to a solution containing 22% glycerol for cryoprotection before flash freezing in liquid nitrogen.

Data for the N74D apo crystals were collected on a RDI CMOS_8M detector at Advanced Light Source (ALS) beamline 4.2.2 at Lawrence Berkeley National Laboratory. Data were processed to 2.9 Å using XDS [27] and indexed in hexagonal space group P6 with unit cell dimensions *a, b* = 91.7 Å and *c* = 57.2 Å, and one CA molecule in the asymmetric unit. Initial phases were solved via molecular replacement with Phaser [28,29], using the coordinates of WT native full-length CA (PDB ID: 4XFX) [26] as a starting model, with all solvent molecules removed. The resulting model was refined using REFMAC5 [30].

Data for the N74D CA/PF74 complex crystals were collected on a Dectris Pilatus3 6M detector at Advanced Photon Source (APS) beamline 23-ID-D at Argonne National Laboratory. Data were processed to 2.65 Å using XDS [27,31] and indexed in hexagonal space group P6 with unit cell dimensions *a, b* = 90.4 Å and *c* = 55.7 Å, and one CA molecule in the asymmetric unit. Initial phases were solved via molecular replacement with Phaser [28,29], using the coordinates of WT native full-length CA in complex with PF74 [26] (PDB ID: 4XFZ) as a starting model, with all ligands and solvent removed. The resulting model was refined using REFMAC5 [30]. PF74 was built into the model using a difference Fourier map calculated in the absence of ligand.

The N74D CA apo and PF74 complex models were each improved through several iterative rounds of model building and refinement, using Coot [32,33] and REFMAC5 [30], respectively. The final models were validated using MOLPROBITY [34]. The final structure factors and coordinates have been deposited into the Protein Data Bank (PDB) and are available under accession codes 7MN0 for the N74D CA apo structure and 7MKC for the N74D CA/PF74 complex structure. Data collection, processing, and refinement statistics are provided in Table S1.

### 2.11. Quantification and Statistical Analyses

Statistical analyses were performed using unpaired *t*-tests. Sample numbers, number of replicates, and *p* values are indicated in corresponding figure legends. Quantification of western blot band intensities was performed using ImageJ. For all experiments, means and standard deviations were calculated using GraphPad Prism 7.0c (San Diego, CA, USA).

## 3. Results

### 3.1. HIV-1-N74D Exhibits Defective Infectivity in Primary CD4^+^ T Cells

**Figure 1:**
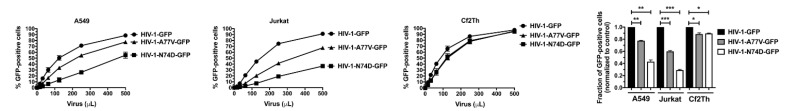
HIV-1-N74D exhibits an infectivity defect in human cell lines but not in dog cell lines. Human lung A549 cells, human Jurkat T cells, or dog thymus Cf2Th cells were challenged with increasing amounts of the indicated p24-normalized WT and mutant HIV-1 viruses. Infectivity was determined at 48 h post-challenge by measuring the percentage of GFP-positive cells. Experiments were repeated three times and a representative experiment is shown. * indicates *p*-value < 0.005; ** indicates *p*-value < 0.001; *** indicates *p*-value < 0.0005 as determined by using the unpaired *t*-test.

To test the role of capsid-CPSF6 interactions during the infection process, we challenged dog and human cell lines with HIV-1 viruses containing the capsid mutations N74D and A77V (both of which prevent capsid interactions with CPSF6). Infectivity of wild-type and mutant HIV-1_NL4-3_∆env, pseudotyped with vesicular stomatitis virus G (VSV-G) envelopes expressing green fluorescent protein (GFP) as an infection reporter, were normalized using p24 levels. HIV-1-N74D-GFP viruses showed a defect on infectivity when compared to wild-type viruses when infecting the lung human cell line A549 or the Jurkat T cell line (Figure 1). HIV-1-A77V-GFP viruses showed a lesser defect when compared to HIV-1-N74D-GFP viruses. By contrast, the infectivity defect of HIV-1-N74D-GFP viruses was not observed in the canine cell line Cf2Th, which do not express TRIM5α orthologues (Figure 1). 

**Figure 2:**
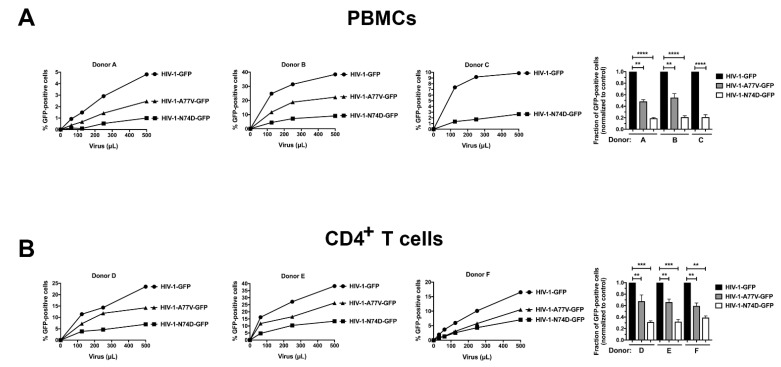
HIV-1-N74D exhibits an infectivity defect in primary PBMCs and CD4^+^ T cells. Human primary PBMCs (**A**); or purified CD4^+^ T cells (**B**) from healthy donors were challenged with increasing amounts of p24-normalized HIV-1-GFP, HIV-1-N74D-GFP, or HIV-1-A77V-GFP. Infectivity was determined at 72 h post-challenge by measuring the percentage of GFP-positive cells. Experiments were repeated three times per donor, and a representative experimental result is shown. Statistical analysis was performed using an intermediate value taken from the infection curves (**right panel**). ** indicates *p*-value < 0.001; *** indicates *p*-value < 0.0005; **** indicates *p*-value < 0.0001 as determined by using the unpaired *t*-test.

Next we tested whether these infectivity defects are present in human primary cells. As shown in Figure 2A, HIV-1-N74D-GFP showed a defect in PBMC infections compared with wild-type HIV-1 in at least three donors. However, HIV-1-A77V-GFP exhibited a moderate infectivity defect when compared with HIV-1-N74D-GFP viruses. Similar results were observed when we challenged human primary CD4^+^ T cells obtained from three independent donors with HIV-1-N74D-GFP and HIV-1-A77V-GFP (Figure 2B). 

### 3.2. Depletion of CPSF6 in Human Primary CD4^+^ T Cells Does Not Affect HIV-1 Infectivity

**Figure 3:**
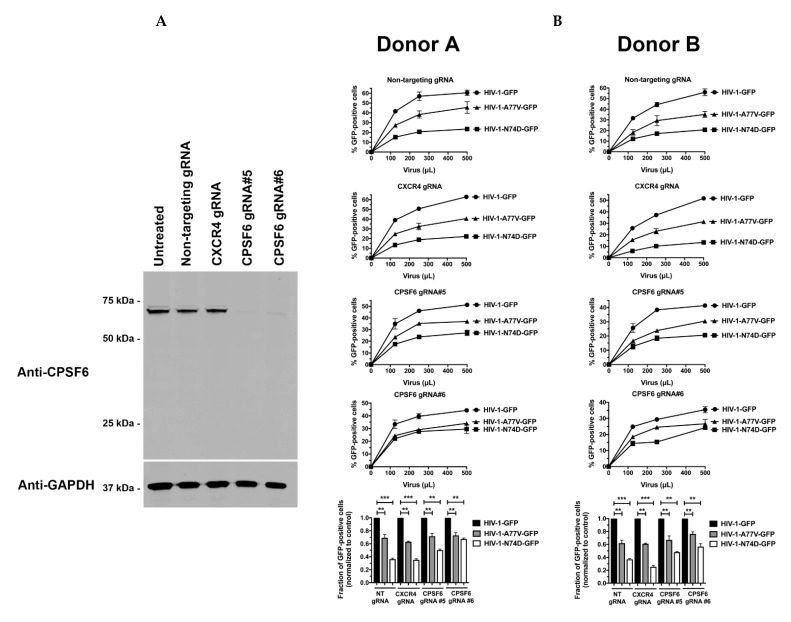
Depleted CPSF6 expression in human primary CD4^+^ T cells does not affect HIV-1 infectivity. (**A**) Human primary CD4^+^ T cells from two different donors had CPSF6 expression knocked out using the CRISPR/Cas9 system, as described in Methods. Briefly, CD4^+^ T cells were electroporated using two different guide RNAs (gRNAs) against CPSF6 (Grna #5 and #6) together with the Cas9 protein. At 72 h post-electroporation, endogenous expression of CPSF6 in CD4^+^ T cells was analyzed by western blotting using an antibody against CPSF6. For controls, a Grna against CXCR4 and a non-targeting Grna were electroporated. Expression of GAPDH was used as a loading control. Similar results were obtained using two different donors, and a representative blot is shown; (**B**) Human primary CD4^+^ T cells depleted for CPSF6 expression were challenged with increasing amounts of p24-normalized HIV-1-GFP, HIV-1-N74D-GFP, or HIV-1-A77V-GFP. Infectivity was determined at 72 h post-challenge by measuring the percentage of GFP-positive cells. Experiments were repeated three times per donor, and a representative experimental result is shown. Statistical analysis was performed using an intermediate value taken from the infection curves (bottom panels). ** indicates *p*-value < 0.001; *** indicates *p*-value < 0.0005 as determined by using the unpaired *t*-test.

To test the role of CPSF6 in HIV-1 infection of human primary cells, we challenged CPSF6-depleted CD4^+^ T cells with wild-type and mutant HIV-1. As shown in Figure 3A, CRISPR-Cas9 ribonucleoprotein complexes (crRNPs) containing the anti-CPSF6 guide RNA (gRNA) #5 and #6 completely depleted the expression of CPSF6 in human primary CD4^+^ T cells. As a control, we also knocked out the expression of CXCR4. Similar to the results above, CPSF6 depletion did not affect wild-type HIV-1 infectivity in human primary cells (Figure 3B). In addition, depletion of CPSF6 did not affect the infectivity of either HIV-1-N74D-GFP or HIV-1-A77V-GFP. These experiments demonstrated that depletion of CPSF6 in human primary cells did not affect HIV-1 infectivity, suggesting that the reduced infectivity of HIV-1-N74D viruses was not due to blocked virus interactions with CPSF6.

### 3.3. Depletion of TRIM5α_hu_ in Human Primary CD4^+^ T Cells Rescues HIV-1-N74D Infectivity

**Figure 4:**
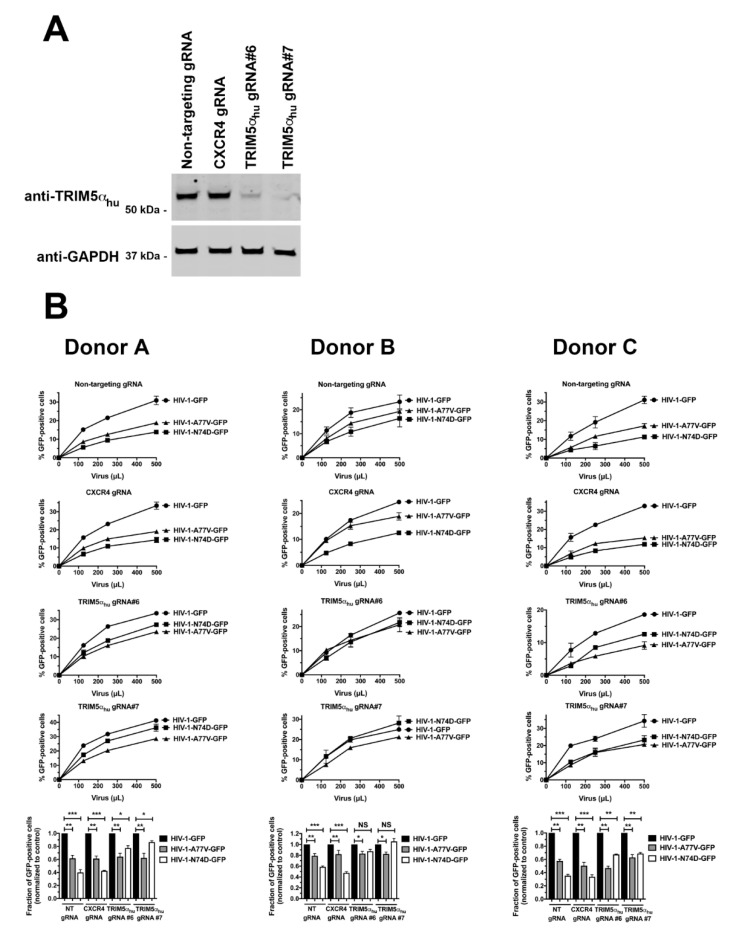
TRIM5α_hu_ depletion in human primary CD4^+^ T cells rescues HIV-1-N74D infectivity. (**A**) Human primary CD4^+^ T cells from three different donors had TRIM5α_hu_ expression knocked out using the CRISPR/Cas9 system, as described in Methods. Briefly, CD4^+^ T cells were electroporated using two different guide RNAs (gRNAs) against TRIM5α_hu_ (Grna #6 and #7) together with the Cas9 protein. At 72 h post-electroporation, the endogenous expression of TRIM5α_hu_ in CD4^+^ T cells was analyzed by western blotting using an antibody against TRIM5α_hu_. For controls, a Grna against CXCR4, and a non-targeting Grna were electroporated. Expression of GAPDH was used as a loading control. Similar results were obtained using two different donors, and a representative blot is shown; (**B**) Human primary CD4^+^ T cells depleted for TRIM5α_hu_ expression were challenged with increasing amounts of p24-normalized HIV-1-GFP, HIV-1-N74D-GFP, or HIV-1-A77V-GFP. Infectivity was determined at 72 h post-challenge by measuring the percentage of GFP-positive cells. Experiments were repeated three times per donor, and a representative experimental result is shown. Statistical analysis was performed using an intermediate value taken from the infection curves (bottom panels). * indicates *p*-value < 0.005; ** indicates *p*-value < 0.001; *** indicates *p*-value < 0.0005; NS indicates not significant as determined by using the unpaired *t*-test.

We and others have previously demonstrated that CypA protects the HIV-1 core from TRIM5α_hu_ restriction in human primary CD4^+^ T cells [17,18]. Therefore, we hypothesized that TRIM5α_hu_ may decrease the infectivity of HIV-1-N74D in human primary cells. To test this hypothesis, we challenged TRIM5α_hu_-depleted human primary CD4^+^ T cells with HIV-1-N74D-GFP. The crRNPs containing the anti-TRIM5α_hu_ Grna #6 and #7 depleted the expression of endogenous TRIM5α_hu_ in human primary CD4^+^ T cells (Figure 4A). As shown in Figure 4B, the depletion of TRIM5α_hu_ considerably rescued HIV-1-N74D-GFP infectivity in CD4^+^ T cells. These results suggested that TRIM5α_hu_ restricted HIV-1-N74D in human CD4^+^ T cells. Interestingly, small infectivity changes were observed for HIV-1-A77V in TRIM5α_hu_-depleted cells, suggesting that this virus is not restricted by TRIM5α_hu._

### 3.4. N74D-Stabilized Capsids Bind to TRIM5α_hu_ but Do Not Interact with Cyp A

**Figure 5:**
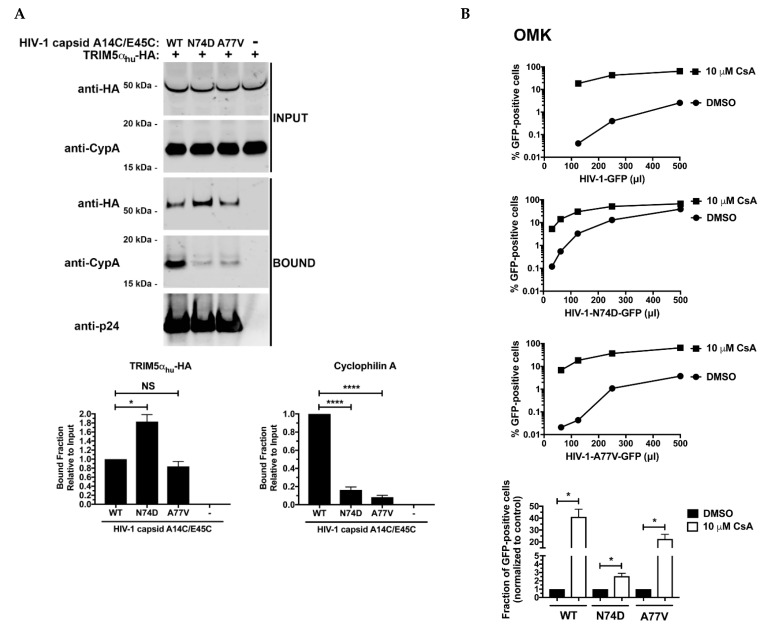

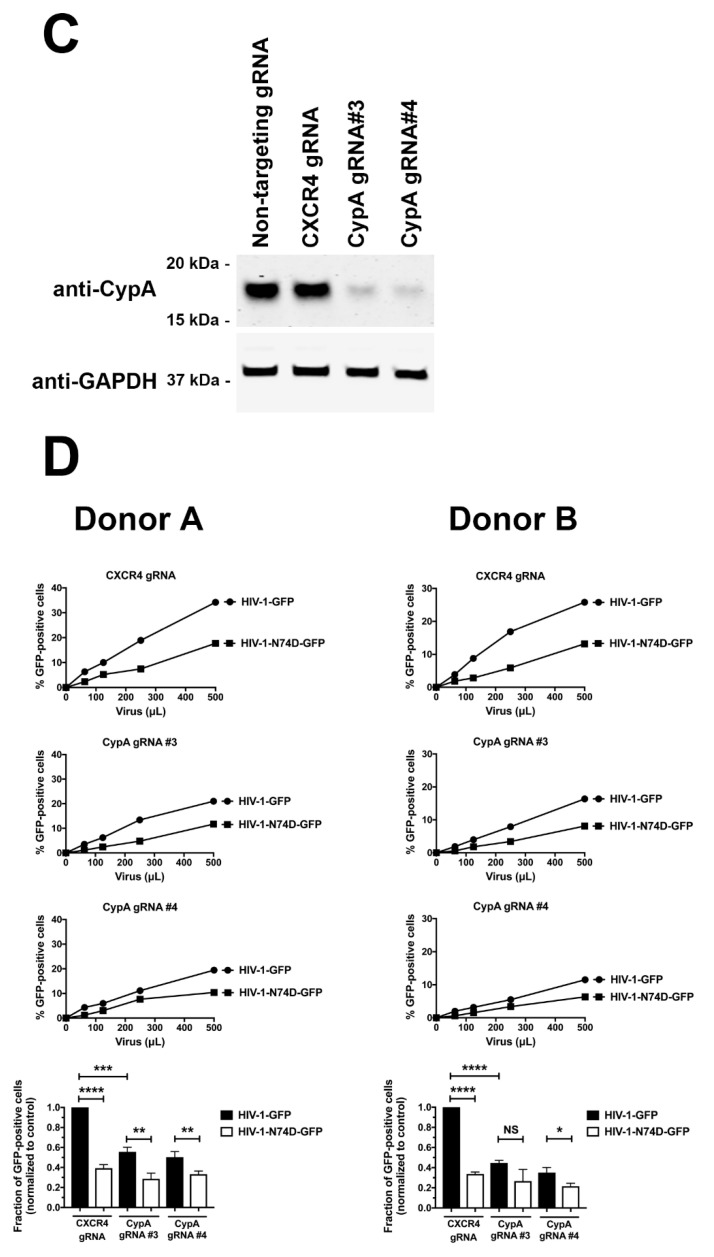
N74D-stabilized capsids bind to TRIM5α_hu_ but do not interact with Cyp A. (**A**) Human 293T cells were transfected with plasmids expressing TRIM5α_hu_- hemagglutinin (HA). Post-transfection (24 h), cells (INPUT) were lysed in capsid binding buffer (CBB) as described in Methods. Cell extracts containing TRIM5α_hu_-HA were then mixed with 10 µL of either stabilized wild-type, N74D, or A77V capsid tubes (5 mg/mL). Mixtures were incubated for 1 h at room temperature. Stabilized HIV-1 capsid tubes were collected by centrifugation and washed twice using CBB. Pellets were resuspended in 1× Laemmli buffer (BOUND). INPUT and BOUND fractions were then analyzed by western blotting using anti-HA, anti-Cyp A, and anti-p24 antibodies. Experiments were repeated three times, and a representative experimental result is shown. The BOUND fraction relative to the INPUT fraction for three independent experiments (with standard deviation) is shown; (**B**) Owl monkey kidney (OMK) cells that endogenously express the restriction factor TRIMCyp were challenged with increasing amounts of the indicated p24-normalized WT and mutant HIV-1 viruses in the presence of cyclosporine A(CsA) or DMSO solvent. Experiments were repeated three times and a representative experiment is shown; (**C**,**D**) Human primary CD4^+^ T cells from two different donors had Cyp A expression knocked out using the CRISPR/Cas9 system, as described in Methods. Briefly, CD4^+^ T cells were electroporated using two different guide RNAs (gRNAs) against Cyp A (gRNA #3 and #4) together with the Cas9 protein. At 72 h post-electroporation, the endogenous expression of Cyp A in CD4^+^ T cells was analyzed by western blotting using an antibody against Cyp A. For controls, a gRNA against CXCR4, and a non-targeting gRNA were electroporated. Expression of GAPDH was used as a loading control. Similar results were obtained using two different donors, and a representative blot is shown (**C**); (**D**) Human primary CD4^+^ T cells depleted for Cyp A expression were challenged with increasing amounts of p24-normalized HIV-1-GFP, or HIV-1-N74D-GFP. Infectivity was determined at 72 h post-challenge by measuring the percentage of GFP-positive cells. Experiments were repeated three times per donor, and a representative experimental result is shown. Statistical analysis was performed using an intermediate value taken from the infection curves (**bottom panels**). * indicates a *p*-value < 0.005; ** indicates a *p*-value < 0.001; **** indicates a *p*-value < 0.0001; and NS indicates no significant difference as determined by unpaired *t*-tests.

Our previous results suggested that TRIM5α_hu_ restricts the infection of HIV-1-N74D viruses. One possibility is that the core of HIV-1-N74D viruses has a decreased affinity for Cyp A making the virus susceptible to restriction by TRIM5α_hu_. To test this hypothesis, we assessed the abilities of TRIM5α_hu_ and Cyp A to bind to N74D-stabilized capsid tubes using a capsid binding assay [22]. As shown in Figure 5A, TRIM5α_hu_ bound with increased affinity to stabilized N74D capsid tubes than to wild-type tubes. Interestingly, TRIM5α_hu_ bound to A77V-stabilized tubes and wild-type tubes in a similar manner. Results from these experiments support the idea that the infectivity defect observed for HIV-1-N74D is due to an increase in TRIM5α_hu_ binding to N74D capsids compared with that to wild-type capsids. We have previously shown that when Cyp A was not expressed in primary CD4^+^ T cells, TRIM5α_hu_ binding to capsid increased [17]; therefore, we tested the ability of Cyp A to bind to N74D-stabilized capsid tubes. As shown in Figure 5A, Cyp A poorly binds to N74D-stabilized capsid tubes, although it bound to wild-type capsid tubes, which is in agreement with a decreased Cyp A binding to N74D capsids [13]. These results showed that N74D capsids were not protected by Cyp A, leading to TRIM5α_hu_ binding and restriction. Interestingly, Cyp A did not bind to A77V-stabilized tubes. Decreased binding of Cyp A to N74D capsid was additionally confirmed in experiments using recombinant Cyp A protein; in this experiment addition of PF74 decreased Cyp A binding to both WT and N74D mutant capsid stabilized tubes (Figure S1).

Our results indicate that HIV-1-N74D viruses do not interact with Cyp A biochemically; therefore, we hypothesized that HIV-1-N74D viruses should be less sensitive to the restriction factor TRIMCyp when compared to wild-type viruses. TRIMCyp binds to the HIV-1 core using its own Cyp A motif and restricts infection [35,36]. To this end, we challenged owl monkey kidney (OMK) cells, which endogenously express TRIMCyp, with increasing amounts of HIV-1-N74D viruses in the presence of cyclosporine A (CsA). If the binding of TRIMCyp to the capsid of HIV-1-N74D viruses is defective, a predicted outcome would be that infection of OMK cells by HIV-1-N74D viruses is more permissive when compared to wild-type HIV-1. As shown in Figure 5B, OMK cells were more susceptible to infection by HIV-1-N74D when compared to wild-type HIV-1. Remarkably, TRIMCyp restriction of HIV-1-N74D viruses is ~2.5-fold when compared to ~40-fold for wild-type HIV-1. These results are consistent with our hypothesis that HIV-1-N74D is defective for their ability to bind Cyp A. Interestingly, TRIMCyp restriction of HIV-1-A77V viruses is closer to wild-type (~20-fold), which agrees with our biochemical experiments.

To further investigate the inability of HIV-1-N74D viruses to interact with Cyp A in human primary CD4^+^ T cells, we infected human CD4^+^ T cells that were knocked out for Cyp A. As shown in Figure 5C and D, the difference of infectivity between WT and mutant viruses decreased upon infection of Cyp A knock-out cells (Figure 5D). These results suggested that in the absence of Cyp A, WT and mutant viruses are equally restricted by human TRIM5α.

### 3.5. The Core of HIV-1 Viruses Bearing the Capsid Change N74D Has a Compromised Stability

**Figure 6:**
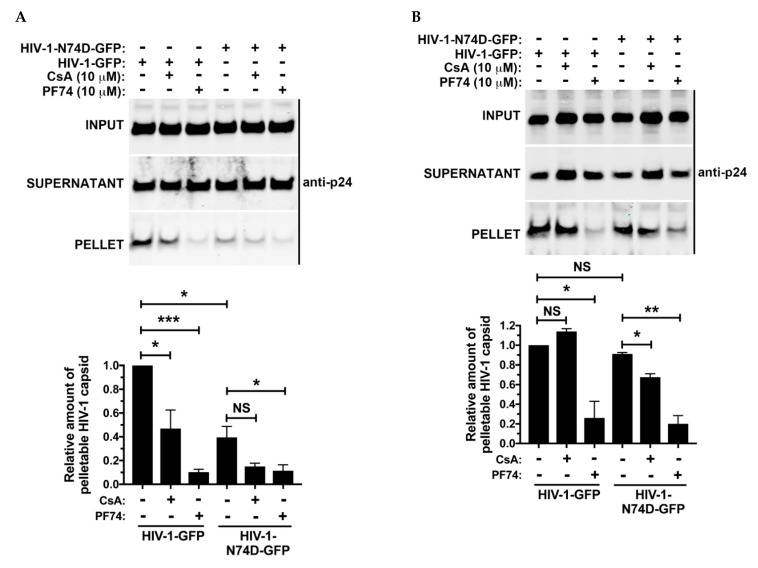
The core of HIV-1 viruses bearing the capsid change N74D has a compromised stability. Wild-type (**A**) or TRIM5α_hu_ knockout (**B**) human cells were infected with p24-normalized WT and mutant N74D HIV-1-GFP viruses (pseudo-typed with VSV-G) at MOI = 2 in the presence of PF74, Cyp A, or DMSO solvent. After incubation for 16 h at 37 °C, cells were harvested and processed using the fate of the capsid assay, as described in methods. INPUT, SOLUBLE, and PELLET fractions of lysed cells were analyzed by western blots using antibodies against the HIV-1 p24 capsid protein. Experiments were repeated three times and a representative experiment is shown. * indicates a *p*-value < 0.005; ** indicates *p*-value < 0.001; *** indicates *p*-value < 0.0005; and NS indicates no significant difference as determined by unpaired *t*-tests.

Given that HIV-1-N74D viruses do not interact with Cyp A, they are susceptible to human TRIM5α [17,18], which accelerates uncoating [19,21,22]. This implies that HIV-1-N74D cores are less stable during infection when compared to wild-type. To test core stability of HIV-1-N74D viruses, we performed the fate of the capsid assay [20]. As shown in Figure 6A, HIV-1-N74D cores are less stable when compared to wild-type during infection. As a control, we utilized PF74, which accelerates uncoating/disassembly of the core [4,37]. Interestingly, the use of CsA further destabilizes wild-type cores, which agrees with the notion that in the absence of CsA, the core is exposed to the uncoating activity of human TRIM5α. These experiments suggested that HIV-1-N74D cores are less stable during infection. To measure the stability of HIV-1-N74D cores in the absence of human TRIM5α, we performed similar experiments using cells that were knocked out for expression of human TRIM5α. As shown in Figure 6B, stability of HIV-1-N74D cores was similar in the presence or in the absence of CsA. As a control, we utilized PF74, which accelerated uncoating of the core. These results showed that in the absence of human TRIM5α HIV-1-N74D cores are as stable as wild-type cores.

### 3.6. Structural Differences between Wild Type and N74D Capsids

**Figure 7:**
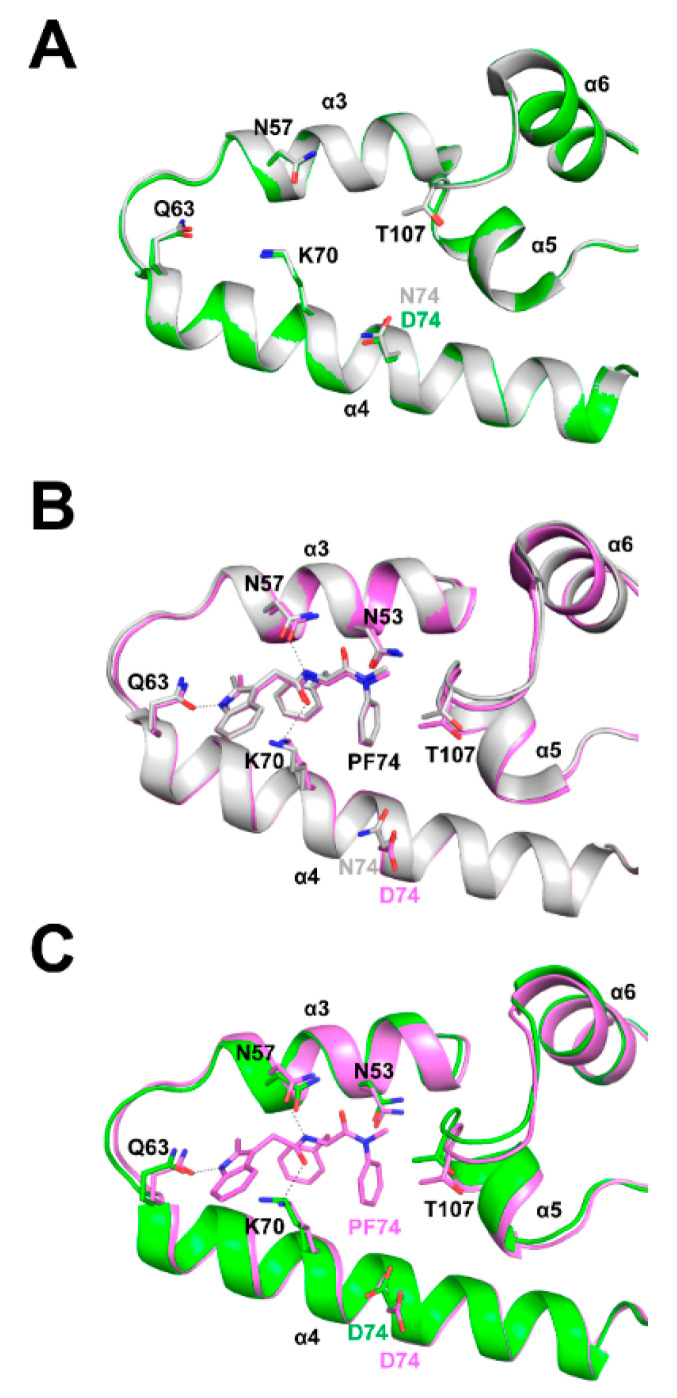
Crystal structures of full-length N74D HIV-1 CA in apo form and in complex with PF74. (**A**) Full-length WT HIV-1 hexameric capsid protein in apo form (PDB ID: 4XFX, gray cartoon) is superposed to the crystal structure of full-length N74D HIV-1 hexameric capsid protein in apo form (PDB ID: 7MN0, green cartoon). Select residues of the PF74 binding pocket are shown as sticks. Residues N74 (WT) and D74 (mutant) are similar in position; (**B**) Full-length WT HIV-1 hexameric capsid protein in complex with PF74 (PDB ID: 4XFZ, gray cartoon) is superposed to the crystal structure of full-length N74D HIV-1 hexameric capsid protein in complex with PF74 (PDB ID: 7MKC, pink cartoon). Select residues and PF74 are shown as sticks, hydrogen bonds with PF74 are shown as black dashed lines. The side chain of residue D74 (mutant) has changed conformation and flips away from PF74 compared to the WT N74 side chain; (**C**) Full-length N74D HIV-1 hexameric capsid protein in complex with PF74 (PDB ID: 7MKC, pink cartoon) is superposed to the crystal structure of full-length N74D HIV-1 hexameric capsid protein in apo form (PDB ID: 7MN0, green cartoon). Select residues and PF74 are shown as sticks, hydrogen bonds with PF74 are shown as black dashed lines. The side chain of residue D74 in the PF74 complex has changed conformation and flips away from PF74 compared to the D74 side chain of the mutant apo structure. CA_CTD_, water, and iodide ions have been removed for clarity.

We then attempted to understand from a structural point of view the reason why HIV-1 capsids bearing the change N74D have decreased affinity for Cyp A. Although the residue N74 is distantly located from the Cyp A binding loop, a mutation such as N74D could impact Cyp A binding by causing an overall structural shift in the assembled HIV-1 core. To explore whether N74D affects the conformation of the capsid hexamer, we have solved crystal structures of full-length N74D hexameric capsid proteins in apo form and in complex with PF74 (Figure 7). First, we compared full-length WT and N74D hexameric capsid proteins in apo form (Figure 7A). Superposition of these structures shows that residues N74 (in WT capsid protein) and D74 (in mutant capsid protein) are in similar positions (Figure 7A).

We have previously shown that the small molecule GS-CA-1 restores the ability of N74D to bind CPSF6 [38], suggesting that the single unit of capsid protein in an assembled core can sample different conformations. These conformational changes translate into a change in the shape of the core, a process known as core breathing [26,39]. To investigate whether the difference between wild-type and N74D capsid conformations occurs during core breathing, we used the small molecule PF74. To this end, we compared the structures of full-length wild-type and N74D hexameric capsid proteins when in complex with PF74. Surprisingly, the wild-type (N74) and mutant (D74) side chains have very different conformations when the capsid protein is in complex with PF74 (Figure 7B). These results suggest that capsid proteins bearing the change N74D are sampling different conformations when compared to wild-type. These different conformations could explain the inability of N74D capsids to bind Cyp A. In agreement, when we compared crystal structures of full-length N74D hexameric capsid protein in apo form and N74D hexameric capsid protein in complex with PF74, we also saw that the D74 residue changes its conformation upon PF74 binding. (Figure 7C). Overall, the change of N74D structure when compared to wild-type could potentially explain the loss of Cyp A binding.

## 4. Discussion

Here we have shown that the infectivity defect observed for HIV-1-N74D viruses in CD4^+^ T cells is due to its inability to interact with Cyp A, which exposes the viral core to TRIM5α_hu_ binding and restriction. These results concur with the idea that Cyp A plays an important role in the protection of the HIV-1 core in the early stages of infection [17,18], and indicate once again that Cyp A may be essential in the early stages of HIV-1 infection to ensure protection of the core from restriction factors or cellular conditions that may affect infection. These results contrast with the current notion that the infectivity defect of N74D is due to its loss of CPSF6 binding [1]. Although capsids bearing the N74D change do not interact with CPSF6, the infectivity defect that HIV-1-N74D viruses exhibit is due to a decrease in Cyp A binding with a concomitant gain of TRIM5α_hu_ binding, which restricts infection. Cell-based infection assays showed that HIV-1-N74D is less sensitive to TRIMCyp when compared to wild-type viruses, suggesting that the core of HIV-1-N74D viruses have a defect binding TRIMCyp, which agrees with our biochemical results. In addition, previous findings suggest that HIV-1-N74D viruses are not affected by the expression levels of Cyp A in macrophages [13]. The combination of our binding and cell-based assays suggested that N74D capsids are defective for binding to Cyp A.

While an intact HIV-1 core has more than ~1200 binding sites for Cyp A, the actual number of sites occupied by Cyp A during an infection is not known; however, it is reasonable to think that binding of one or two Cyp A molecules per hexamer would be sufficient to prevent the binding of restriction factors such as TRIM5α_hu_ by steric hindrance. So theoretically, only two Cyp A molecules per hexamer would be needed to ensure that infection is productive. Interestingly, residue N74 in the capsid structure is distantly located from the Cyp A binding loop, suggesting that an overall structural shift may be occurring to prevent Cyp A binding. An alternative explanation is that the N74D mutation may affect core breathing, and consequently, inhibit the binding of Cyp A to the core [26]. One of the implications of this study is that interactions between Cyp A and capsid mutants should be considered when trying to understand HIV-1 infectivity defects involving human primary cells since mutations in different locations of the capsid may impact capsid conformation and binding to cellular factors.

To further understand the inability of N74D to bind Cyp A, we compared the structures of wild-type and N74D full-length hexameric capsid proteins in complex with PF74. Our results suggested that both capsid proteins, when in complex with PF74 are sampling different conformations in the assembled core, which may be related to the inability of N74D to bind Cyp A. It is possible that although the Cyp A binding loop is distant from N74D, the change of capsid conformation could trigger an overall shift in the conformation of the core, changing its ability to bind Cyp A.

The infectivity defect of HIV-1-A77V viruses was not very pronounced when compared to HIV-1-N74D viruses. In addition, depletion of TRIM5α_hu_ did not rescue the infectivity defect of HIV-1-A77V. In agreement, A77V stabilized capsid tubes did not show an increase in binding to TRIM5α_hu_, but lost binding to Cyp A. One possibility is that HIV-1-A77V viruses are defective for a different reason, which agrees with the experiments showing that HIV-1-A77V viruses can replicate in CD4^+^ T cells when compared to HIV-1-N74D [16].

Taken together, these results suggest that TRIM5α_hu_ may be working together with TRIM34 to reduce HIV-1-N74D infectivity. We demonstrated previously that TRIM5α proteins can form higher-order self-association complexes, which are essential for TRIM5α-based restriction of retroviruses [40,41]. In addition, these studies showed that TRIM5α proteins can also form higher-order complexes with TRIM orthologs such as TRIM34 and TRIM6 [42]. It is possible that TRIM5α_hu_ forms higher-order complexes with TRIM34 in order to bind and restrict HIV-1-N74D viruses, as we have previously suggested [43].

These results highlight the importance of HIV-1 core-Cyp A interactions during productive HIV-1 infection and indicate that Cyp A is an essential cofactor for HIV-1 replication in human primary CD4^+^ T cells.

## Figures and Tables

**Table 1:** RNA guides.

Synthetic RNA/Gene Target	Guide #	Sequence	Catalog Number (Dharmacon)
Edit-R Synthetic tracrRNA	n/a	n/a	U-002005-50
Edit-R crRNA Non-targeting Ctrl #3	3	n/a	U-007503-20
CXCR4 crRNA	1	GAAGCGTGATGACAAAGAGG	Custom sequence
CPSF6 crRNA	5	GGACCACATAGACATTTACG	CM-012334-05
CPSF6 crRNA	6	ATATATTGGAAATCTAACAT	Custom sequence
TRIM5alpha crRNA	6	AAGAAGTCCATGCTAGACAA	Custom sequence
TRIM5alpha crRNA	7	GTTGATCATTGTGCACGCCA	Custom sequence

## Data Availability

Not applicable.

## Supplementary Materials

The following supporting information can be downloaded at: https://www.mdpi.com/article/10.3390/v14020363/s1, Figure S1: Recombinant CypA binds with decrease affinity to N74D-stabilized capsid tubes; Table S1: X-ray data collection and refinement statistics for the full-length N74D HIV-1 CA apo and PF74 complex crystal structures.
