# Supplementary material for: TRIM5α Restriction of HIV-1-N74D Viruses in Lymphocytes Is Caused by a Loss of Cyclophilin A Protection
Source: Viruses. 2022 Feb 10;14(2):363. doi: 10.3390/v14020363 (PMC8879423; doi:10.3390/v14020363)
Supplement: Supplementary file 1 [file viruses-14-00363-s001.zip › viruses-1490188-supplementary.pdf]

Table S1. X-ray data collection and refinement statistics for the full-length N74D HIV-1 CA apo and PF74 complex crystal structures.

	**N74D CA apo**	**N74D CA/PF74**
	PDB ID: 7MN0	PDB ID: 7MKC
**Data collection**	Beamline ALS 4.2.2	Beamline APS 23-ID-D
Wavelength (Å)	1.000030	1.03317
Resolution (Å)	2.90 (2.90-3.08)[a]	2.65 (2.65-2.81)[a]
Space group	P6	P6
Cell dimensions		
a, c (Å)	91.7, 57.2	90.4, 55.7
Observed reflections	69,089	41,748
Unique reflections	6,190	7,562
Redundancy	11.1 (10.6)	5.5 (5.3)
Completeness (%)	99.8 (99.4)	98.3 (96.9)
R_{meas}[b]	0.129 (1.22)	0.100 (0.82)
$CC_{1/2}$	99.9 (71.5)	99.8 (68.8)
Avg I/σ	16.3 (2.0)	11.4 (2.0)
**Refinement statistics**		
Resolution (Å)	45.87-2.90	45.38-2.65
No. of reflections (working)	5,866	7,190
No. of reflections (test)	323	366
R_{work}[c]	0.219	0.183
R_{free}[d]	0.274	0.242
Overall B value (Å^2)	84.0	67.0
Wilson B value (Å^2)	70.1	60.0
Ramachandran plot (%)[e]		
Favored	93.4	94.5
Allowed	6.6	5.5
Disallowed	0	0
All-atom clashscore	4	3
RMSD Bond length (Å)	0.019	0.019
RMSD Angle (°)	1.904	1.960

[a] Values in parentheses are for the outer resolution shell.

[b] $R_{meas} = \Sigma_{hkl} \sqrt{\frac{n}{n-1}} \sum_{j=1}^{n} |I_{hkl,j} - < I_{hkl} > | \;/\; \Sigma_{hkl} \Sigma_j I_{hkl,j}$.

[c] $R_{work} = \Sigma_{hkl} |F_{obs} - F_{calc}| \;/\; \Sigma_{hkl} |F_{obs}|$.

[d] $R_{free} = R_{work}$, except 5% of the data excluded from the refinement.

[e] Evaluated by MolProbity (Chen *et al.*, Acta Cryst. D66, 2010, 12-21).

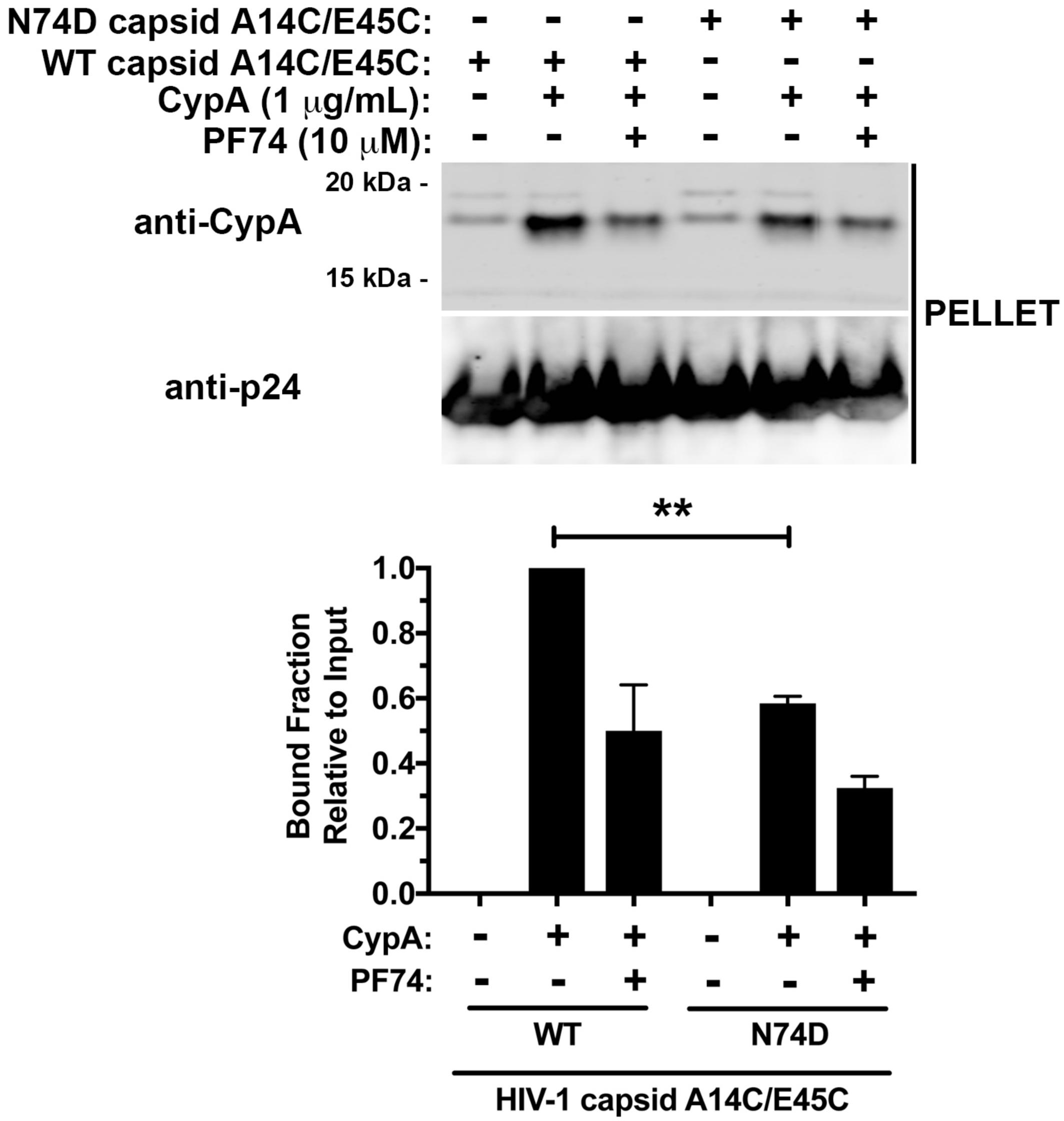

Figure S1. **Recombinant CypA binds with decrease affinity to N74D-stabilized capsid tubes.** Recombinant CypA protein was diluted in capsid binding buffer (CBB) to a final concentration of 1 µg/mL. 40 µL of this mixture were mixed with Laemmli buffer and used as INPUT (not shown). 300 µL of this mixture were mixed with 10 µl of either stabilized wild-type, or N74D capsid tubes (5 mg/ml) in presence of PF74 (10 µM) or DMSO. Mixtures were incubated for 1 h at room temperature. Stabilized HIV-1 capsid tubes were collected by centrifugation and washed twice using CBB. Pellets were resuspended in 1× Laemmli buffer (BOUND) and then analyzed by western blotting using anti-Cyp A and anti-p24 antibodies. Experiments were repeated three times, and a representative experimental result is shown. The BOUND fraction relative to the INPUT fraction for three independent experiments (with standard deviation) is shown. ** indicates P-value < 0.001 as determined by using the unpaired t-test.
